# The association between health-promoting-lifestyles, and socioeconomic, family relationships, social support, health-related quality of life among older adults in china: a cross sectional study

**DOI:** 10.1186/s12955-022-01968-0

**Published:** 2022-04-20

**Authors:** Xiao Zheng, Yaqing Xue, Fang Dong, Lei Shi, Shujuan Xiao, Jiachi Zhang, Benli Xue, Yi Qian, Hong Zhu, Qiang Man, Chichen Zhang

**Affiliations:** 1 School of Public Health, Southern Medical University, Guangzhou, Guangdong China; 2 School of Health Management, Southern Medical University, No. 1023, South Shatai Road, Baiyun District, Guangzhou, Guangdong China; 3 Institute of Health Management Southern Medical University, Guangzhou, Guangdong China; 4 Department of Health Management, Nanfang Hospital, Southern Medical University, Guangzhou, Guangdong China; 5 School of International Education, Southern Medical University, Guangzhou, Guangdong China

**Keywords:** Older adults, Health-promoting-lifestyles, Social support, Health-related quality of life, Structural equation modeling

## Abstract

**Objectives:**

Lifestyles, accounting for 53% in determining death, play a vital role in improving the health of older adults. Thus, this study aimed to explore the influencing factors of the health-promoting-lifestyles and interaction mechanisms among older adults.

**Methods:**

A total of 8526 elders were selected by a three-stage stratified random cluster sampling method. Socioeconomic status, family relationships, social support, health-related quality of life (QOL), and health-promoting-lifestyles (HPLP) of older adults were assessed with the Social Support Rating Scale, the short form 36 health survey (SF-36) and Health-Promoting Lifestyle Profile. A structural equation model (SEM) was conducted to test the direct and indirect association between influencing factors with HPLP.

**Results:**

In this study, there were 4901 older adults who were empty nesters, and 3625 were non-empty nesters. Of all respondents, the average QOL score of older adults was 62.28 ± 16.51, average social support score was 78.06 ± 7.50. The HPLP score of older adults was 105.9 ± 19.6, and the average score of subscales was 2.5 ± 0.5, which was at the medium level. Social support had a positive and direct effect on HPLP of older adults (total effect, 0.34). Meanwhile, social support mediated the relationship between socioeconomic (total effect, 0.17), QOL (total effect, 0.33) and HPLP. Family relationships had a small indirect effect on HPLP via social support (0.01).

**Conclusions:**

Social support is the strongest influencing factor in the health-promoting-lifestyles among older adults, followed by socioeconomic, health-related quality of life and family support. Thus, maintaining higher social support was important to improve the HPLP of older adults.

## Background

China has entered an era of an aging society at the end of the twentieth century with an extended average life expectancy and a lower birth rate affected by a massive rapid economic development [1]. Specifically, National Bureau of Statistics of China shows that 17.3% population aged 60 years and above in 2017 [2]. Older adults could always face a high prevalence of physical and mental disease, and population aging is projected to have a profound effect on societies, underscoring the fiscal and political pressures that the health care, older adults, and social protection systems of many countries are likely to face in the coming decades [3–5]. Therefore, finding an effective way to improve the health condition of older adults has been more and more prominent for an aging society.

World health organization (WHO) point out over 53% of deaths around the world were caused by adverse behavioral lifestyles [6]. Some chronic diseases were known as behavior-related diseases, such as the occurrence of hypertension is closely associated with poor health-promoting-lifestyles [7]. And due to the unhealthy lifestyles, increased prevalence of physical disorders, nursing shortage, low income, and social isolation, older adults would feel lonely and depression. Based on the important of healthy behaviors and changes in disease structure, it is importance to provide support to older adults to help establish and maintain a healthy lifestyle [8].

Some researchers have confirmed that age, monthly income, sources of income, education level, marital status, and the number of children were influencing factors of older adults’ health-promoting-lifestyles [9]. For instance, Cheng [9] found that there was a statistic difference between health-promoting-lifestyles and the aspects of age, monthly income, sources of income, and community health education. And health-promoting lifestyle has a significant positive correlation with self-rated health of older adults. Additionally, Xu [10] found that marital status, income and education were major factors impeding the local popularization of health-promoting-lifestyles of older adults. Chen [11] claimed the number of children should be paid more attention as well as marital status and education degree. Analyzing the above variables, they could be divided into three latent variables, such as family factors, social factors, and socioeconomic factors.

Who is the most effective backup of health-promoting-lifestyles among older adults? There was no research focusing on this question. Therefore, this study aimed to discuss who was the most effective factor of health-promoting-lifestyles and relationships between family, social support and socioeconomic among older adults. Cultural values of family and filial piety are the backbone of traditional elder care systems in China [12]. Hence, children could be the fundamental guardian of the health among the elderly.

**Fig. 1:**
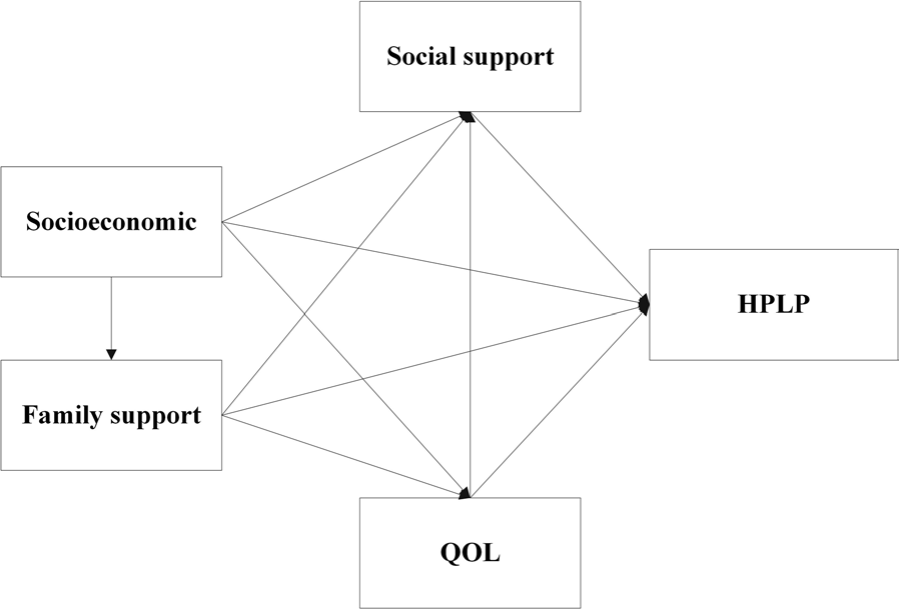
Theoretical model of this study

There were pieces of evidence that health inequalities may widen with age [13]. A study indicated older adults’ need for care impose self-restrictions due to their poverty, which were harmful to health and limit the quality of life [14]. A Study of Chen found that neighborhood support networks and community facilities contribute significantly to low-income elders’ depression [15]. But some prospective studies showed that people with depression who had poor social support have worse outcomes in terms of symptoms, recovery, and social functioning. Social support emerged as an important role in the physical and mental health of older adults [16], and good quality of life was positively associated with older adults’ social support. Based on this, we found socioeconomic, family support, social support, and QOL were associated with each other, and all would predict the health-promoting-lifestyles of older adults. Few studies focused on the relationship of those different factors on how older adults live a health-promoting-lifestyles. Structural equation modeling (SME) including the estimation of models with regressions among continuous latent variables or factors is commonly justified in the social sciences because of its ability to impute relationships between latent variables from observable variables [17, 18]. We used SME to construct a theoretical model that contains all the relationships between socioeconomic, family relationships, social support, QOL, and HPLP (Fig. 1).

To do so, this study has the following specific objectives. First, the health-promoting-lifestyles of older adults would be identified. Secondly, the health-promoting-lifestyles of different participants would be compared. Finally, SME would be used to explore the direct and indirect factors of older adults’ health-promoting-lifestyles. The results will provide evidences for the improvement of health for older adults.

## Methods

### Sample

This study was conducted in 11 cities in Shanxi province. Of 9000 individuals were contacted, 8526 completed the questionnaires. The effective response rate was 94.7%. A three-stage stratified random cluster sampling method was used to select older adults in each city. First, two district (county) were randomly selected in each city. Next, two communities (village) were randomly selected in each district using a random-number table, which can draw random sampling scientifically. Third, a residential district was randomly chosen from each selected community using the same method. All older adults aged over 60 were included in this study, and if there were no enough elderly, another residential district was selected to replace it. Older adults aged 60 years and above, who could communicate in and understand Chinese and had no cognitive disorders were eligible for the study.

All older adults were interviewed in an isolated room using a structured questionnaire by trained postgraduate students. The Ethical Committee of Shanxi Medical University approved the study protocol. The investigation was conducted after the informed consents of all participants.

### Instruments

#### Social demographic characteristics including

1. Socioeconomic status was categorized as follows: education (elementary education, secondary education, higher education), Pre-retirement occupation (jobless, migrate workers, farmer, commercial and service laborers, dealer, worker, laborers of company, laborers of public institution), Source of income (social relief, children, personal and spouse, pension), monthly income (no, < 1000 yuan, 1000 yuan~, 3000 yuan~).
2. Family relationships included marital status (widowed, divorced, spinsterhood, married) relationship with children (no children or bad relationship versus, good relationship), spouse relationship (no spouse or bad relationship versus, good relationship).
3. Social support The Social Support Rating Scale was used to measure the social support of the participants. It consists of 10 items with a total score ranging from 12 to 66; a higher score indicates a higher level of social support [19]. The Cronbach’s α of this scale was 0.94.
4. health-related quality of life (QOL) The short form 36 health survey (SF-36) is a 36-item valid quality of life questionnaire with 2 sub-scales on physical health and mental health. The Physical Component Summary (PCS) and Mental Component Summary (MCS) were used in this study [20]. The Chinese version of the SF-36 has been extensively validated. The Cronbach’s α of the SF-36 was 0.83 in this study.
5. Others Empty nest, Gender, Age, residence.

#### Health-promoting lifestyle profile (HPLP)

The Chinese version of the Health-Promoting Lifestyle Profile was developed and tested in Taiwan [21]. We have adopted the HPLP to measure the lifestyle of empty nesters in six dimensions including self-realization, health responsibility, physical activity, nutrition, interpersonal relations, and stress management, with a total of 42 items. The score for each dimension was the summative score of its all items, and the HPLP score was the sum of the scores of all dimensions, ranging from 42 to 168. Based on the single split for the scale, the health-promoting-lifestyles can be divided into 3 levels: high (3–4 points), medium (2–3 points), and low (1–2 points). In the studied population, the internal consistency of the scale was high, overall Cronbach’s α was 0.91, with all 6 dimensions ≥ 0.59.

### Statistical analysis

Firstly, descriptive analyses were performed to describe the actuality of social demographic characteristics and HPLP for respondents. Secondly, A two-sample *t*-test, single factor analysis of variance, and Kruskal–Wallis H test were used to compare the health-promoting-lifestyles across different subgroups of older adults by SPSS24.0. Finally, the identified factors associated with health-promoting-lifestyles were included in structural equation modeling (SEM) with path analysis using AMOS22.0. In this study, the maximum likelihood estimation method was used to evaluate the suitability of the model. Covariance between measured variables was set to the system default value of 1, with no controlling factors. Model fit was based on thresholds for the goodness-of-fit index (GFI), adjusted goodness-of-fit index (AGFI) and root mean square error of approximation (RMSEA) which belongs to absolute fitness index, normed fit index (NFI), and root mean square residual (RMR). The GFI, AGFI, and NFI all range from 0 to 1, with values above 0.9 indicating an acceptable fit. An RMR and an RMSEA with values approximating 0.05 indicate a good fit [22].

## Results

In this study, there were 4901 older adults who were empty nesters, and 3625 were non-empty nesters, 53.6% older adults were ranged from 60 to 70 years, about 49.7% were males and 50.3% were females, 5543 elders lived in rural. The average QOL score of older adults was 62.28 ± 16.51, MCS was 63.57 ± 17.45, PCS was 0.99 ± 18.18, average social support score was 38.06 ± 7.50. Of all respondents, the total score of HPLP was 105.9 ± 19.6 and the average score of subscales was 2.5 ± 0.5. The health-promoting lifestyles of older adults were in the medium level; the score of dimensions were nutrition (2.9), followed by self-realization (2.6), interpersonal relations (2.5) stress management (2.5) and physical activity (2.3), the lowest score was health responsibility (2.2).

The HPLP scores for different types of demographics were shown in Table 1. There were statistically significant differences in the HPLP for demographic characters of Empty nest, gender, age, resident, education, marital status, Pre-retirement occupation, Source of income, monthly income (*P* < 0.05), expect for working (Table 1).

**Table 1:** Factors associated with HPLP among older adults

Characteristics	N (%)	$\overline{\mathrm{X} }$±S	t/F/H	*P*
*Empty nest*			− 3.86	< 0.001
Empty nest	4901(57.5)	105.3 ± 19.7		
Non-empty nest	3625(42.5)	106.9 ± 19.5		
*Gender*			2.09	0.037
Males	4239(49.7)	106.4 ± 19.8		
Females	4287(50.3)	105.6 ± 19.4		
*Age*			38.08	< 0.001
60 years ~	4570(53.6)	106.9 ± 19.6		
70 years ~	2956(34.7)	106.2 ± 19.4		
80 years ~	1000(11.7)	101.0 ± 19.7		
*Residence*			− 19.81	< 0.001
Rural	5543(65.0)	102.9 ± 18.8		
Urban	2983(34.9)	111.7 ± 19.9		
*Education*			503.78^a^	< 0.001
Primary education	5352(62.8)	102.5 ± 19.0		
Secondary education	2753(32.3)	110.9 ± 18.5		
Higher education	421(4.9)	118.1 ± 21.9		
*Marital status*			176.83^a^	< 0.001
Widowed	2067(24.2)	101.4 ± 19.2		
Divorced	184(2.2)	106.9 ± 18.7		
Spinsterhood	236(2.8)	101.3 ± 21.9		
Married	6039(70.8)	107.7 ± 19.4		
*Pre-retirement occupation*			99.89	< 0.001
Jobless	607(7.1)	102.8 ± 20.6		
Migrate workers	431(5.1)	105.6 ± 20.6		
Farmer	4179(49.0)	101.4 ± 18.4		
Commercial and service laborers	152(1.8)	110.9 ± 16.5		
Dealer	335(3.9)	110.8 ± 18.9		
Worker	906(10.6)	109.3 ± 18.3		
Laborers of company	732(8.6)	114.1 ± 19.5		
Laborers of public institution	1184(13.9)	114.4 ± 19.2		
*Working*			0.05	0.963
No	7732(90.7)	105.9 ± 19.5		
Yes	794(9.3)	105.9 ± 21.2		
*Source of income*			495.07^a^	< 0.001
Social relief and others	668(7.8)	99.3 ± 20.7		
Supply from children	2983(34.9)	102.9 ± 18.5		
Personal and spouse	2209(25.9)	103.9 ± 18.9		
Pension	2666(31.3)	112.7 ± 19.4		
*Monthly income*			598.60^a^	< 0.001
No	3060(35.9)	101.4 ± 18.9		
< 1000 yuan	2269(26.6)	103.3 ± 18.5		
1000 yuan~	2171(25.5)	110.3 ± 18.7		
3000 yuan~	1026(12.0)	116.5 ± 20.3		
*Relationship with children*			− 2.88	0.004
No children or bad	1067(12.5)	104.4 ± 19.7		
Good	7459(87.5)	106.2 ± 19.6		
*Spouse relationship*			− 2.26	0.024
No spouse or bad	766(8.9)	104.5 ± 19.9		
Good	7760(91.0)	106.1 ± 19.6		

^a^Kruskal–Wallis H test

**Fig. 2:**
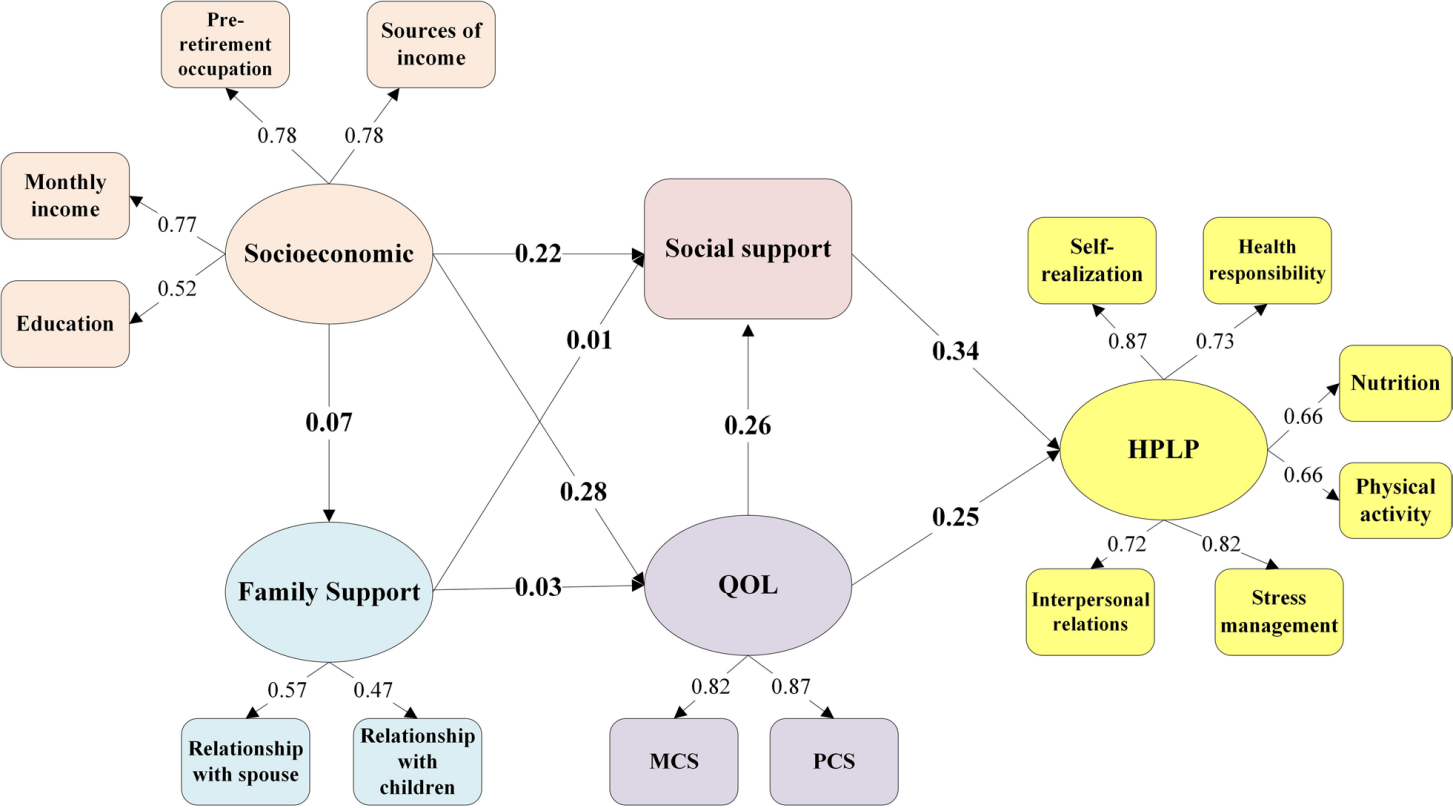
The structural equation model of HPLP among older adults

SEM was employed to test the influencing factors of HPLP among older adults. In the model, the skewness coefficient and kurtosis coefficient of all latent variables were less than 3 and 8. The hypothesized model consisted of five latent variables and 15 observed variables (Fig. 2). In the model, model fit index of GFI was 0.95, and AGFI was 0.93, NFI was 0.94, and an RMSEA was 0.06. The assessment suggests that the hypothetical model provides a good fit for the data.

The model results were showed in Fig. 2. Social support had a positive and direct effect on HPLP of older adults (Total effect, 0.34). Socioeconomic had a direct effect on family relationships (Direct effect, 0.07), QOL (Direct effect, 0.28), and social support (Direct effect, 0.22, *P* < 0.001); QOL had a direct effect on social support (Direct effect, 0.26). Meanwhile, social support mediated the relationship between socioeconomic (Total effect, 0.17), QOL (Total effect, 0.33) and HPLP. Family relationships had a small indirect effects on HPLP (Total effect, 0.01).

Moreover, the greatest absolute value of standardized total effects was from social support (0.34), followed by socioeconomic (0.33), QOL (0.25), and family support (0.01). Table 2 presented the total, direct and indirect effects of the variables on HPLP.

**Table 2:** Standardized total, direct and indirect effect of the variables on HPLP

Latent variable	Direct effect	Indirect effect	Total effect
Social support	0.34	/	0.34
Socioeconomic	/	0.17	0.17
QOL	0.25	0.08	0.33
Family support	/	0.01	0.01

The stability of the model of influencing factors among elders’ HPLP was verified by taking empty nest status, gender, and age as the classification indices. The results indicated that six models all adequately represented the data structure, the model of the operating factors for older adults’ HPLP was steady (Table 3).

**Table 3:** Adaptive index values for group models

Fitness index	Empty nesters	Non-empty nesters	Males	Females	60 years ~	70 years ~
GFI	0.94	0.94	0.95	0.94	0.95	0.93
AGFI	0.93	0.92	0.93	0.92	0.93	0.92
RMSEA	0.03	0.06	0.05	0.06	0.05	0.06
NFI	0.91	0.90	0.92	0.90	0.92	0.90

## Discussion

In the study, the average QOL score of older adults was 62.28 ± 16.51, average social support score was 78.06 ± 7.50. Of all respondents, the total score of HPLP was 105.9 ± 19.6 and the average score of subscales was 2.5 ± 0.5. The HPLP of older adults were in the medium level. We found that social support was the top effective factor of HPLP among older adults. In addition, socioeconomic status, family support, and QOL would eventually affect older adults’ HPLP via social supports.

The activity theory of aging proposes that successful aging occurs when older adults stay active and maintain regular interactions with others in society [23]. It takes the view that the aging process is delayed and the quality of life is enhanced when old people remain socially active [24]. The study of Yang [25] found that family support and social supports can affect the HPLP of older adults. And Lu found that social support showed moderating effect on the relationship between depression and HPLP among empty nesters [26]. Higher social support showed that older adults had more social activity, which could enhance the communication between older adults. Through communication, older adults could learn from each other, which has a positive effect on the improvement of their nutrition and health responsibility [27]. Hence, improving the social support of older adults was an important way to enhance their healthy lifestyles. Another finding of this study was the mediating role of social support in the relationship between socioeconomic status, QOL, family support and HPLP. The indirect effect of socioeconomic status, QOL on HPLP via social support was found to be very strong, suggesting that social support played a critical role in affecting the relationship between socioeconomic status QOL and HPLP.

In addition, this study found that for older adults who have higher QOL, their HPLP were better. Some Studies confirmed that mental health was associated with behaviors, such as exercise, nutrition and self-realization [28]. Mental health and physical health portend older adults have a positive attitude, which could help them to solve unhealth events in their life [29]. And people with higher QOL, have more time to improve their HPLP [30].

Also, socioeconomic [31, 32] is an economic and sociological combined total measure of work experience and individual or family economic and social position of people, based on income, education, and occupation. The education level is an indication of social capital, physiological capital, and economic capital. Occupation is an indication of responsibility, physical activity, and health risks. Income is an indication of one’s consumption ability, housing, and the ability of access to medical resources. We found there was a positive significance between observational variables of socioeconomic status such as education, monthly income, and health-promoting-lifestyles. But there was no direct effect between socioeconomic and HPLP. Socioeconomic status would eventually affect older adults’ HPLP via social supports, family relationships and QOL. The result was like the results of current research, socioeconomic status would affect HPLP through neighborhood or social psychological factors [33]. The higher the socioeconomic status, the richer the social resources are available to the individuals [20]. In previous research, Yang found socioeconomic status such as age, education and the source of income, family relationships such as family structure were influencing factors of elders’ social activity [34]. Older adults who have higher socioeconomic status, and they will have more time to participate in social activity, which was positively associated with their HPLP.

Older adults live with their families for the longest time, but the results found that the effect of family relationships was lower than social support on older adults’ HPLP. Older adults are facing a multifaceted transform of their role in the family [35]. For instance, children go out to work and older adults become empty nesters and have received no care from their children. Older adults become grandparents, and grandchildren become the focus of the family [36]. The changes in family structure led to the weakening of the family function for older adults [37]. Previous studies have suggested that close relationship with children was a major factor in improving behavioral lifestyles among the elderly [8]. The results of this study indicated that family support was associated with HPLP of older adults via social support. But the family relationships would be influenced by older adults’ socioeconomic status.

This study had some limitations. First, the data and relevant analyses presented here were conducted using subjective questionnaires, information bias cannot be ruled out. Second, this was a cross-sectional study, the study results failed to determine causal relationships among the variables, so it is crucial to follow up with longitudinal research.

## Conclusion

This study explored the influencing factors of HPLP and the interaction mechanism among older adults. The results revealed that social support had a direct effect on HPLP among older adults. The impact of socioeconomic status, quality of life and family relationships on HPLP were mediated by social support. Based on the results of this study, the government and the community should organize social activities for older adults and provide them more social support. Meanwhile, At the family level, providing necessary financial support and emotional attention is essential to improving health-promoting-lifestyles of older adults.

## Data Availability

The datasets used and/or analysed during the current study are available from the corresponding author on reasonable request.
